# Psychological outcomes of extended reality interventions in spinal cord injury rehabilitation: a systematic scoping review

**DOI:** 10.1038/s41393-024-01057-7

**Published:** 2025-01-09

**Authors:** Samuel David Williamson, Anders Orup Aaby, Sophie Lykkegaard Ravn

**Affiliations:** 1 Specialized Hospital for Polio and Accident Victims, Rødovre, Denmark; 2 Department of Psychology, University of Southern Denmark, Odense, Denmark

**Keywords:** Quality of life, Psychology

## Abstract

**Study design:**

Systematic scoping review.

**Objectives:**

Extended reality (XR) is becoming a recognisable tool for assisting in spinal cord injury (SCI) rehabilitation. While the success of XR mediated interventions is often evaluated based on improvements in physical and functional performance, the present systematic scoping review aimed to identify and synthesize evidence on reported psychological outcomes of XR interventions in SCI rehabilitation. In doing so, we aimed to contribute towards an adaptation of XR that is meaningful for individuals living with SCI.

**Methods:**

Seven bibliometric databases were systematically searched. Included studies needed to be peer-reviewed, test structured and targeted XR interventions in an adult (≥ 16 years) SCI population, and assess any psychological construct. Individual double-screening against a pre-defined eligibility criteria was performed. Data from the included studies were extracted, tabulated, and analysed.

**Results:**

A total of 964 unique studies were initially identified. 13 studies were included in the analysis. The psychological outcomes most frequently quantified were depression, self-esteem, and anxiety. Among other things, qualitative evidence suggests VR-based interventions provided enjoyment, relaxation, and a source of positive distraction.

**Conclusion:**

Immersive XR interventions in SCI rehabilitation have been positively evaluated, both qualitatively and quantitatively, based on the psychological outcomes of participants. While further research is needed, we find immersive XR to be an emerging treatment option with promise for maintaining and improving psychological health during SCI rehabilitation.

## Introduction

In recent years, extended reality (XR) technologies have become favourable healthcare tools not only for surgical planning [1] and medical training [2], but also to improve rehabilitation outcomes in various neurological populations [3–5]. XR technology also holds great promise in treating common phobias [6] and various mental health disorders [7]. While a number of adverse factors might hinder engagement with the technology, such as simulator sickness [8], muscular fatigue [9], and the training required for both participants and clinicians [10], it remains important to explore the potential of XR technology in rehabilitation settings to guide recommendations for clinical practice.

XR is an umbrella term to describe virtual reality (VR), augmented reality (AR), and mixed reality (MR) devices [11]. These three technologies can be differentiated by the scale of their immersion and realism. In brief, VR describes a product of computer modelling that allows for an individual to interact with a three-dimensional simulated environment [12]. In comparison, AR encourages interaction with an authentic, real-world environment overlayed with digital information. MR combines the interactivity of VR with the authenticity of AR so that users can manipulate superimposed images to a far greater degree than in AR [13].

As their names imply, the unifying factor between these technologies is their ability to alter perception, reconstruct the user’s experience, and simulate reality in a controlled setting. Consequently, XR has been appraised as a means to generate an embodied, first-person perspective of experiences and environments that would otherwise prove difficult to encounter as an individual sustaining a life-altering, traumatic injury [14]. One such example is spinal cord injury (SCI), a population that has benefited from XR in a number of rehabilitation contexts, improving physical health outcomes such as balance [15–18], pain [19, 20], and functional performance [21]. However, in line with our contemporary understanding of rehabilitation [22], addressing psychological outcomes is a fundamental component of SCI rehabilitation [23]. As such, it is important to also explore psychological outcomes of XR interventions in SCI rehabilitation. While reviews within the SCI literature have assembled evidence on psychological outcomes before [24–26], no such example accounts for XR technologies outside of VR, thereby excluding the growing AR and MR literature. Equally, no comparable review requires that eligible VR interventions be delivered via fully immersive, 360° head-mounted devices. Moreover, the rapid development and adoption of this technology in rehabilitation settings has led to increased research activity, highlighting the need for updated reviews. However, to our knowledge there exists no current systematic scoping review that specifically focuses on assembling evidence on psychological outcomes, such as quality of life, mental health, and coping, attributed to XR interventions within SCI rehabilitation. Therefore, this review aims to contribute towards an adaptation of XR that is effective, useable, and meaningful for individuals in SCI rehabilitation. To achieve this, the review identifies, critically appraises, and synthesizes the available literature based on an updated evidence base.

## Methods

This systematic scoping review was performed in compliance with the Preferred Reporting Items for Systematic Reviews and Meta-Analyses Protocols (PRISMA) 2020 expanded checklist [27]. The protocol associated with this review was pre-registered in the Open Science Framework as of 23^rd^ January 2024 (DOI: 10.17605/OSF.IO/FSC9R).

### Information sources and search strategy

The search string comprised two blocks containing search terms either synonymous or associated with a) SCI or b) XR. The choice of these terms was informed by prior author experience and a thorough reading of relevant systematic reviews concerned with the use of XR as a treatment option within neurological rehabilitation. Embase (via OVID, 1947-present), Medline (via OVID, 1946-present), APA PsycInfo (via OVID, 1806- present), Web of Science (1900-present), Scopus (1823-present), CINAHL (via EBSCOhost, 1982-present), and the Cochrane Central Register of Controlled Trials (1940-present) were chosen as databases to process our search string. Subject headings and medical subject headings (MeSH terms) were adjusted to accommodate the unique indexing of each database. Where new subject headings or MeSH terms were introduced to the string, so too were their free-text equivalents. The free-text component of the string was entered identically in all databases. Within-block searches were combined using the Boolean Operator “OR”, while between-block searches were combined using the Boolean Operator “AND”. This process was repeated several times to ensure consistency before the final searches were performed. The final search string features as Supplement 1 in our supplementary materials.

### Eligibility criteria

To warrant inclusion, studies had to meet all requirements of our predefined eligibility criteria. This constituted of studies being written in English, Danish, Swedish, or Norwegian, being peer-reviewed, and presenting original data, in full, from a structured and targeted XR intervention performed in a rehabilitation context. This necessarily excluded material published as a review, book chapter, conference abstract, protocol, editorial, letter, online registrations, or dissertation. XR interventions were only valid where software was delivered via a fully immersive, 360° head-mounted device. Studies had to have recruited adults (≥16 years of age only) with SCI. Where SCI participants were included in mixed samples, the target population must account for ≥50% of the total population, or otherwise make SCI results distinguishable from the whole. Similarly, where psychological domains were included as part of a broader, multidimensional scale, it was required that domains of interest were reported independently from the total score. Studies had to assess a psychological construct such as, but not limited to, quality of life, mental health, and coping strategies. Further, these constructs must not have been represented by a biomarker alone (e.g., serotonin levels to evaluate depression). No specifications were set for study design, outcome or publication year.

### Screening procedure

The first 100 records were independently double-screened in Covidence [28] between three reviewers at the title and abstract level. Disagreements were discussed and resolved internally. Screening continued until a unanimous verdict was reached on all records. Where the content of a record was deemed relevant, but the format was in breach of our eligibility criteria, that record was tagged and documented. Having completed the screening of all remaining records at the title and abstract level, those forwarded were independently double-screened at the full-text level between four reviewers. Reasons for exclusion were documented at this level. Disagreements were again discussed and resolved internally or via the assistance of a clinician where necessary. Where information relevant to the decision-making process was unclear, the first author contacted the study’s corresponding author(s) for clarification.

### Additional searches

Google Scholar and PEDro were used to help identify relevant studies not found by our database searches. Five combinations of between-block search terms (e.g., spinal cord injur* + extended realit*) were entered into Google Scholar. The first 50 results of each search, sorted initially by relevance and later by date, were screened in a conservative manner by the first author. Five searches were conducted on 26^th^ February 2024, and a further five searches were conducted on 3^rd^ June 2024. Those considered eligible at this title-and-abstract adjacent level were imported into Covidence at the full-text screening level for independent assessment by the first author and a research assistant. PEDro was searched for relevant clinical trials on 16^th^ February 2024, and later on 3^rd^ June 2024, using the search term “spinal cord injury” exclusively. All potentially eligible studies were subject to the same treatment as those found via Google Scholar. All tagged studies excluded on account of their format (i.e., otherwise relevant reviews, book chapters, protocols, conference materials etc.), underwent a process of forward and backward citation tracking by the first author. Google Scholar assisted the former, while the latter was performed manually. Again, studies considered relevant at this approximated title-and-abstract screening level were subject to a full-text screen in Covidence by two independent reviewers. Newly included studies underwent citation tracking until no relevant additional studies surfaced. Once the screening process was concluded and the final dataset was confirmed, all included studies were searched in Google to ensure that no correctional or retracted information was published post-hoc.

### Data extraction

To ensure accurate reporting, all relevant data from included studies were independently extracted by two reviewers. Information pertaining to authorship, year of publication, country of origin, sample size (n), sample characteristics (age [range and mean/median]), sex [percentages]), injury characteristics (injury level, injury completeness, and time since injury), setting, study design, intervention type, outcome(s), and main results were all formatted into a standardized table. Information provided upon request from corresponding authors was inserted into a final column, titled ‘other’. The extraction table features as Supplements 2 and 3 in our supplementary materials.

### Synthesis methods

**Fig. 1:**
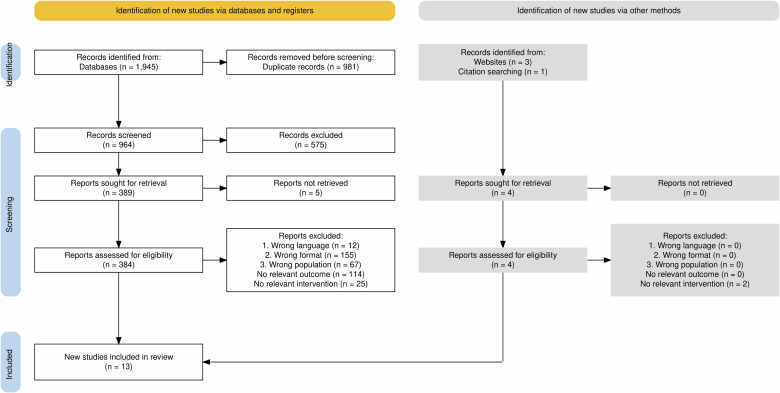
Flow diagram of the study selection process. This PRISMA-compliant diagram displays the systematic and unsystematic search process, screening, and reasons for exclusion, with final (unsystematic) searches conducted on June 3rd, 2024.

To visualize the screening process, the first author completed a flow diagram compliant with the PRISMA Statement [29] (Fig. 1). Using the extraction table for reference, the results were narratively synthesized by the first author and subsequently organized into distinct chapters and subchapters based on feedback from the remaining authors.

### Risk of bias assessment

As per the protocol, the Cochrane Risk of Bias Version 2 [30] and the Risk-of-Bias in N-of-1 [31], designated to randomized controlled trials (RCTs) and N-of-1 designs respectively, were the only tools pre-specified to perform quality assessment, while all remaining designs, such as case control and qualitative studies, were appropriately matched by the first and second author to a formalized risk of bias assessment tool. Five additional tools were subsequently chosen: the CASP Qualitative Studies Checklist [32]; two JBI Critical Appraisal Checklists - one designed for Case Reports [33] and another for Quasi-Experimental Studies [34]; and two NHLBI Quality Assessment Tools - one designed for Case Series Studies [35] and another for Before-After (Pre-Post) Studies with no Control Group [36].

## Results

### Study flow

The combined sum of our seven database searches totalled 964 unique studies, 384 of which were screened at the full-text level. Studies retrieved via database searches were excluded based on ineligible format (*n* = 155), outcome (*n* = 114), population (*n* = 67), intervention (*n* = 25), and language (*n* = 12). Four additional studies were identified via unsystematic searches and forward and backward citation tracking, two of which were excluded due to an eligible intervention. Therefore, a total of 13 studies were included via a combination of systematic and unsystematic searches. Figure 1 visualizes the study flow in greater detail.

### Study characteristics

The included studies were heterogenous in terms of research design. Designs included case reports (*n* = 4), within-subject randomized crossover trials (*n* = 2), feasibility studies (*n* = 2), pre-post longitudinal cohort studies (*n* = 1), quasi-experimental studies (*n* = 1), non-randomized, controlled pilot studies (*n* = 1), pilot randomized controlled trials (RCTs) (*n* = 1), and single-case experimental designs (*n* = 1). The total number of participants assessed across the 13 included studies was 111. Men (*n* = 87, 78.4%) were overrepresented in comparison to women (*n* = 24, 21.6%). The age range of participants spanned from 26 to 70 years old. Included studies were published between 2000 and 2024, with nine published in the last four years, and 12 published in the last eight years. Studies were conducted across several continents. Of the three types of XR interventions, only VR was used in the included studies.

### Narrative synthesis

Qualitative and quantitative findings from the 13 included studies are presented in separate chapters. Qualitative data is presented under subheadings described in terms of broad themes or states. This approach was taken to prevent the misalignment of nuanced quotes with diagnosable mood and affective disorders. Quantitative data was organized into subcategories for those psychological outcomes measured by more than one study, i.e., depression, anxiety, and self-esteem. An additional subchapter compiles psychological outcomes measured by one study alone.

#### Qualitative evidence

##### Enjoyment

Three studies reported the enjoyment participants experienced when engaging with VR interventions [37–39]. VR therapeutic singing was described as ‘interesting’, ‘different’, ‘cool’, ‘good’, and ‘fun’ [37]. Navigation within a 3D model of a semi-independent living space was considered both ‘fun’, and a ‘nice aspect’ during rehabilitation from a traumatic injury [38]. Another VR intervention simulated walking on various natural surfaces such as grass, sand, and stone, optimised with thermal and tactile stimulation. In response, the case participant remarked feeling as though he was standing up and walking for the first time in 30 years [39].

##### Well-being and relaxation

Two studies described the effect of VR on well-being and relaxation [40, 41]. Assessed over 14 months, one case participant reported that VR walking visualizations positively impacted on well-being and quality of life [40], in addition to providing valuable moments of relaxation [40]. Another case participant performed assisted-walking with a novel orthopaedic device combined with a 360° hotspot-based VR display [41]. Post-intervention, the participant reported feeling relaxed and observed improvements in his overall well-being and mood having ambulated through simulated mountainous terrain [41].

##### Mindfulness

One case report demonstrated how a VR dialectical behavioural therapy (DBT) programme assisted participants in successfully practicing mindfulness [42]. During the sessions, participants listened to a combination of mindfulness training audio tracks and were lay in a supine position to simulate the experience of gently floating down a river.

##### Positive distraction and motivation

Four studies focused on positive distraction and motivation as outcomes of immersive VR interventions in SCI rehabilitation [37, 38, 41, 43]. One VR singing intervention was described as a temporary escape that provided a brief distraction from injured-associated complications [37]. Another individual felt momentarily detached from reality and all its self-described ‘mundanity’ [37]. Similarly, one participant operating an arm ergometer and pedalling in VR reported feeling more present, unaware of their pain, and not focusing on their body [43]. Another indication of the distractive quality of VR is evident from one participant’s temporal disorientation during the intervention [43]. Additionally, three studies described participants either experiencing greater willpower [41] or feeling newly motivated [38, 43] to engage in rehabilitation due to the novelty [38] of VR optimisation.

##### Confidence

Two studies reported improvements in confidence following VR interventions [38, 41]. One focus group attendee anticipated that VR tours would expediate the process of gaining independence in new environments for individuals with SCI [38]. Similarly, VR was appraised as a helpful tool for simulating experiences prior to their execution in real-world settings [38]. Another participant conjectured that VR sessions might represent an accessible alternative to activities requiring specialized equipment and facilities, such as a gym workout [38]. Additionally, in testing an integrated gait-training and VR protocol, one paraplegic case participant cited ‘slight improvements’ in self-confidence post-intervention [41].

##### Reduced inhibition and presumed isolation

One study, evaluating a VR-based therapeutic group singing intervention, observed a reduced sense of inhibition among participants [37]. One participant likened VR to a ‘mask’ shielding the user from peer critique, while another attributed the phenomenon to not seeing themselves during the activity [37]. However, one participant felt less connected to the group members in VR than in a prior teleconferencing condition [37]. Additionally, participants reported that VR-based therapeutic singing reduced social cues when performed in a group setting [37].

#### Quantitative evidence

##### Depression

The most frequently measured psychological outcome was depression, evaluated by six studies [42, 44–48]. When depressive symptomatology was assessed shortly after the intervention, positive results were observed across studies. VR interventions were shown to reduce depressive scores on the Patient Health Questionnaire 8 [46] and the Patient Health Questionnaire 9 [47], irrespective of order of assignment [46] or participation in an interactive or passive VR condition [47]. Similarly, two case participants of a VR-mediated DBT programme demonstrated consistent reductions in pre-to-post depressive mood over a combined six sessions. [42]. Longer-term assessments of VR participation on depressive mood in SCI lack consensus. One case report observed consistent weekly improvements on the Hamilton Depression Test following a 6-week protocol of arm and leg cycling optimized by VR. The SCI participant scored 33 at week one, determined as ‘severe depression’, down to 5 at week 6, recognized as a ‘normal’ psychological state [48]. Comparatively, non-significant improvements in PHQ-8 depression scores were observed by Lakhani et al., irrespective of whether participants experienced the VR condition before or after the control condition [46]. Two studies reporting depression as a secondary outcome observed similarly mixed findings. Participants of a 12-month neurorehabilitation programme supplemented by immersive VR consistently registered ‘good’ scores on the Beck Depression Inventory [45]. Contrastingly, Austin et al. observed no significant differences in depressive mood among individuals participating in immersive and non-immersive VR conditions, as measured by the 21-item Depression, Anxiety and Stress Scale (DASS-21) [44].

##### Anxiety

Anxiety was measured by four studies [42, 44, 46, 48]. Consistent reductions in Spielberger State-Trait Anxiety Inventory scores were reported for two participants undergoing VR DBT [42]. Longitudinal improvements in anxiety are evidenced in two further studies. Over 6 weeks of VR-optimized exercise, Hamilton Anxiety Test scores declined significantly and in line with improvements in somatic functioning [48]. Elsewhere, participants in a pre-post study demonstrated reduced feelings of anxiety across all three sessions, during which natural environments were simulated through VR [46]. However, one randomized cross-over pilot study reported nonsignificant differences in DASS-21 anxiety scores between head-mounted 3D VR and non-immersive 2D presentations [44].

##### Self-esteem

Two studies assessed self-esteem [37, 45]. Engagement in a therapeutic group singing intervention, supported by commercial and custom VR, demonstrated modest, positive improvements on the self-esteem domain of the Psychosocial Impact of Assistive Devices Scale [37]. Though no statistics are formally reported, all eight participants of a prospective longitudinal study combining VR with visuo-tactile feedback experienced ‘good’ scores on the Rosenberg Self-Esteem Scale over 12 months [45].

##### Other psychological outcomes

Several psychological outcomes were measured by just a single study. Improvements on the Positive and Negative Affect Schedule were observed in response to both an interactive and a passive VR walking condition [47]. Both participants of a combined protocol of brain-computer interface and VR training considered the intervention to be mentally demanding, despite experiencing varying levels of frustration, as reported on the NASA Task Load Index [49].

Two case participants evaluated VR DBT using a graphic rating scale which quantified the intensity with which seven primary emotions were experienced [42]. For one case participant, ratings of sadness, fear, and anger reduced after each of four sessions, feelings of guilt were relieved in half of the sessions, and no effect was reported on shame, disgust, or joy. Comparatively, the other participant experienced decreased fear and shame, while sadness, anger, and guilt were experienced to a greater extent [42]. One study reported no observable impact of 3D head-mounted VR, as opposed to a laptop display, on stress, as measured by the DASS-21 [44]. While no statistical data were reported, a brain-machine interface-based gait protocol supplemented by VR was characterized by ‘good’ scores on the World Health Organization Quality of Life Assessment Instrument-Bref [45].

### Risk of bias

The most significant RoB results are presented below. A full summary is included as Supplement 4 in our supplementary materials. In no RCT [43, 44, 46] were participants blinded to the study condition. Where participants could accurately distinguish the control from the intervention, and where participants self-reported changes in psychological outcomes, we believed it feasible that responses ‘could’ conform to the assumed expectation or desire of the researchers. Similar concerns about the lack of researcher blinding were raised during the assessment of two non-controlled studies [45, 49]. Insufficient sample size was considered a risk of bias for Donati et al. [45] and Ferrero et al. [49]. Concerns for generalizability are evidenced elsewhere, with the inclusion of four single participant case studies [39–41, 48], and a case series design featuring two participants. Patterns were also observed among the quasi-experimental studies [37, 47] where treatment conditions of compared groups were unclearly juxtaposed outside of the current experimental setting. Both Tamplin et al. [37] and Trost et al. [47] failed to measure all relevant outcomes several times both preceding and proceeding the intervention in order to meaningfully disregard alternative explanations for the intervention’s effect.

## Discussion

This systematic scoping review consolidated and synthesised the existing literature concerning the reported psychological outcomes of participating in XR interventions during SCI rehabilitation. A total of 13 studies were included, all of them testing VR interventions. Among others, qualitative assessments speak to the ability of VR to provide enjoyment [37–39], facilitate mindfulness [42], and engender confidence [38]. In terms of quantitative evidence, depression [42, 44–48], anxiety [42, 44, 46, 48], and self-esteem [37, 45] were the most frequently measured and improved outcomes. Increased mental demand [49], high frustration levels [49], and communication difficulties in group administration [37] were reported as barriers to engagement.

### Study findings in context

In the current review, we identified no psychological outcomes measured in response to AR or MR interventions in SCI rehabilitation. Prior attempts to synthesize XR interventions within the stroke [50], acquired brain injury [51], and aging population [52] literature have similarly found no evidence for the use of MR in a rehabilitation context. Likewise, systematic reviews within the healthcare literature have also failed to identify evidence of rehabilitative AR interventions [53]. Nevertheless, in a recent cross-disciplinary review of XR applications across fields such as phobia treatment, pain distraction, and addiction training, AR interventions were more common in physical/mental rehabilitation than in any other discipline [54]. More specifically, evidence from the stroke literature has demonstrated the positive effect of AR on many of the same psychological domains included in the present review, including anxiety [55], self-confidence [55], frustration [55], relaxation [55], happiness [55], stress [56], and motivation [55, 57]. Since AR is validated as a treatment option for other neurological populations, future research might begin to explore the benefits of the technology on such outcomes as depression, anxiety, and self-esteem during treatment within SCI.

Comparatively, the treatment of psychological disorders using immersive VR has received considerable attention. A recent scoping review finds 16 studies evaluating immersive VR interventions for depression care [58], while another systematic review determined that, out of all clinical disorders and impairments, those diagnosed with anxiety were the most frequent benefactors of immersive VR treatment [59]. In our qualitative findings, VR simulations of natural environments were regularly perceived as ‘relaxing’ by participants. Similar findings were observed in a recent systematic review investigating the relaxing quality of VR for people diagnosed with mental health disorders [60]. A significant proportion of Riches et al.’s [60] included studies that used head-mounted technology also observed improvements in the most quantified domains of the present review, namely, depression [61, 62] and anxiety [61–68]. These findings are supported by a comparable review conducted in the general population, which found immersive VR to offer relaxation in times of collective stress [69].

SCI participants also referenced a feeling of transcendence in immersive VR environments, both from the physical discomforts of their own body and from preconceived notions of its limitations. This phenomenon is evidenced in the wider literature, most notably in systematic reviews reporting the effect of head-mounted VR for distracting from and alleviating chronic [70, 71] and acute [71] pain. Similar reports are found in a report of individuals sustaining burn injuries where, on average, the duration of time spent focusing on their pain during physical therapy decreased significantly from 60 mm to 14 mm on a 100 mm visual analogue scale [72]. When communication using head-mounted VR was mediated by an avatar, a number of participants of the present review reported feeling disconnected from fellow group members. Conflicting evidence from the wider literature might indicate this to be a context-specific finding. In a group therapy intervention for individuals with clinical depression, participants appreciated the anonymity provided by avatars, allowing them to confide in their therapists in an honest and uninhibited manner [73]. Another study finds that when physical and demographic characteristics were anonymized by an avatar, healthcare users were more likely to seek out and interact with VR mental health treatments [74].

### Limitations

Self-care measures, such as the Spinal Cord Independence Measure [75], refer more generally to one’s physical capacity to perform daily tasks such as feeding, bathing, and dressing. To avoid conflating this data with psychological health assessments, multicompartmental outcome measures presenting a total self-care score, and without publishing independent results for the psychological domain, were not considered for inclusion [76–81]. While this decision was consistent with the study protocol, it alludes to a more general issue of determining what could and could not be considered a psychological construct. While it was agreed to exclude biomarkers as indicators of psychological adjustment, such as the dysregulation of norepinephrine signifying heightened anxiety levels, our protocol was intentionally non-committal in its description of psychological constructs, suggesting quality of life, mental health, and coping only as examples of eligible outcomes. In considering a broad range of outcomes, our review captures both momentary mood states observed during or immediately after the intervention and at no point thereafter, in addition to sustained psychological outcomes assessed over multiple time points. As such, readers are encouraged to consider the context of each study, particularly in regard to temporal and environmental factors, when comparing study outcomes.

Due to the heterogeneous nature of the study designs included in our analysis, the risk of bias assessment of our 13 studies was completed using six different tools, each judging quality against its own distinct and uniquely described criteria. This diversity complicates the process of drawing cross-sample quality assessments. Nevertheless, we remain steadfast in the belief that to hold contrasting study designs up to one, fixed standard would be imprecise and, ultimately, uninformative.

### Avenues for future research

The current review contains three studies that derive psychological outcomes from a single trial of VR [37–39]. When interpreting the results of these studies it is important to consider the novelty effect [82], which suggests that, as the originality of a newly prescribed treatment approach subsides, so do its initial effects [83]. While single trial protocols can offer meaningful qualitative evidence concerning momentary mood states [84], it is acknowledged that brief assessments of VR do not account for the possibility of diminishing returns, or the general observation of psychological outcomes as they develop over time. In other population groups, reports of both positive [85] and negative [86] affect are associated with longitudinal XR designs. However, at present there are too few published studies within the SCI literature evaluating the long-term effects of XR interventions on psychological outcomes to provide a meaningful conclusion on the technology’s lasting effects. As such, more longitudinal study designs with follow-up assessments might allow for a richer conversation regarding XR’s lasting impact on psychological health during SCI rehabilitation.

In narrowing our scope to 360° head-mounted XR interventions we necessarily excluded non- [87–89] and semi-immersive [90–96] XR hardware, regardless of the relevance of the participant group. Many such examples are designed to be operated without the need for wearable technologies. A future review might consider comparing non-, semi-, and fully immersive XR interventions on psychological health in an SCI population.

## Supplementary information

Supplement 1. Final search string, conducted on 15th January 2024

Supplement 2. Extraction of study characteristics

Supplement 3. Extraction of intervention and relevant main results

Supplement 4. Risk of bias assessment, including the foremost quality concerns, of the 13 included studies

## Data Availability

All data generated or analysed during this study are included in this published article and its supplementary information files.
