# Supplementary material for: Psychological outcomes of extended reality interventions in spinal cord injury rehabilitation: a systematic scoping review
Source: Spinal Cord. 2025 Jan 9;63(2):58–65. doi: 10.1038/s41393-024-01057-7 (PMC11810788; doi:10.1038/s41393-024-01057-7)
Supplement: Supplementary file 1 — Supplement 1. Final search string, conducted on 15th January 2024 [file 41393_2024_1057_MOESM1_ESM.docx]

| **Population Block** | **Intervention Block** |
| --- | --- |
| Spinal cord injury (SH) wo/e | Virtual reality (SH) wo/e |
| Spinal cord lesion (SH) wo/e | Augmented reality (SH) wo/e |
| Quadriplegia (SH) wo/e | “Virtual realit*” |
| Paraplegia (SH) wo/e | “Augmented realit*” |
| Paralysis (SH) wo/e | “Extended realit*’” |
| Spinal cord injuries (SH) wo/e | “Mixed realit*” |
| Spinal injuries (SH) wo/e |  |
| Spine injury (SH) wo/e |  |
| “Spinal cord injur*” |  |
| “Spinal cord lesion*” |  |
| “Spinal cord trauma*” |  |
| “Spinal injur*” |  |
| “Spinal lesion*” |  |
| “Spinal trauma*” |  |
| “Spine injur*” |  |
| “Tetraplegi*” |  |
| “Paraplegi*” |  |
| “Quadriplegi*” |  |
| “Paraly*” |  |
