# Supplementary material for: Psychological outcomes of extended reality interventions in spinal cord injury rehabilitation: a systematic scoping review
Source: Spinal Cord. 2025 Jan 9;63(2):58–65. doi: 10.1038/s41393-024-01057-7 (PMC11810788; doi:10.1038/s41393-024-01057-7)
Supplement: Supplementary file 2 — Supplement 2. Extraction of study characteristics [file 41393_2024_1057_MOESM2_ESM.docx]

| Author (year) | Country of Origin | Sample  Type (N) | Sample Characteristics | Injury Characteristics | Study Setting | Study Design | Type of Equipment | Study Outcome(s) | Other |
| --- | --- | --- | --- | --- | --- | --- | --- | --- | --- |
| Austin et al. (2021) | Australia | 16 | 16 men (100%) 0 women (0%) Mean age: 54.3 (14.1) | Traumatic: 15 (94%) Non-traumatic: 1 (6%)  Lower thoracic/upper lumbar: 8 (50%) Mid thoracic: 5 (31%) Upper thoracic: 1 (6%) Lower cervical: 2 (13%)  Complete lesion (N=11) & incomplete lesion (N=5).  ASIA A: 10 (63%) ASIA B: 2 (12%) ASIA C: 1 (6%) ASIA D: 3 (19%) | Community sample. Intervention performed in-hospital. | Within-subject randomized cross-over trial. | Oculus Rift headset | The Depression Anxiety Stress Scale (DASS-21) |  |
| Azurdia et al. (2022) | USA | 11 | 5 men (45.45%) 6 women (55.55%)  Mean age: 43.29 ± 17.5 | SCI patients with chronic pain Paraplegia: 2 (18%) Tetraplegia: 9 (82%) | Participants visited the center 3 times. Community sample. Clinical setting | Within-subject, randomized crossover trial, with a concurrent, nested, mixed-methods approach | HTC VIVE HMD | Pain Self Efficacy Questionnaire (PSEQ) Fatigue Assessment Scale (FAS) Patient-reported outcomes |  |
| Chu et al. (2024) | China | 1 | 50-year-old woman | Spinal stenosis and C3–C7 cervical disk herniation. Incomplete quadriplegia. | Clinical sample. Inpatient at a rehabilitation department. | Single subject case report. | Lei Niao  Air Plus VR glasses. | Hamilton anxiety  (HAMA) and Hamilton depression (HAMD) tests |  |
| Donati et al. (2016) | International (Brazil, USA, and Switzerland | 8 | 6 men (75%) 2 women (25%)  Mean age: 31.13 Age range: 26-38 | Paraplegic SCI patients  ASIA A: 7 (87.5%) ASIA B: 1 (12.5%)  Complete lesion: 7 (87.5%)  Incomplete lesion: 1 (12.5%)  Time since lesion (TSL): 3-13 years (mean 7.13 years)  P1: R=T11, L=T10, TSL = 13yrs P2: R=T4, L=T4, TSL = 6yrs P3: R=T10, L=T11, TSL = 5yrs P4: R=T8, L=T8, TSL = 5yrs P5: R=T7, L=T7, TSL = 3yrs P6: R=T4, L=T4, TSL = 8yrs P7: R= T7, L=T5, TSL = 6yrs P8: R=T11, L=T11, TSL = 11yrs | Intervention performed both in-hospital and at-home over 12 months | Pre-post, non-controlled, longitudinal cohort study. | Oculus Rift, Oculus VR | World Health  Organization Quality of Life Assessment Instrument-Bref (WHOQoL-Bref), the Rosenberg Self-Esteem Scale, and the Beck Depression Inventory (BDI) |  |
| Ferrero et al. (2023) | Spain | Total: 12  (SCI: 2) | 2 men (100%) 0 women (0%) Mean age: 56.5 Age range: 51-62 | P1: T4 ASIA C Incomplete  P2: L3 ASIA B  Incomplete | SCI participants recruited from the National Hospital of Paraplegics in Toledo. Intervention performed in-hospital. | Quasi-experimental study | ‘VICE HTC’, assumed to mean  ‘HTC VIVE’ | NASA Task Load Index (NASA-TLX)  Quebec  User Evaluation of Satisfaction with Assistive Technology (QUEST 20) |  |
| Flores et al. (2018) | USA | 2 | P1: 39-year-old man P2: 31-year-old man | P1: C4-5, 2 weeks post-injury  P2: C7, 2 weeks post-injury | Clinical sample. Intervention performed in-hospital | Within-subject design case study. | Oculus Rift DK2 VR goggles | Beck Depression Index (BDI) Medical Fast Screen  The Spielberger State-Trait Anxiety Inventory (STAI-Y)  Graphic Rating Scale (GRS), and a GRS of current ASD/PTSD symptoms  Each patient briefly rated on a scale from 0-10 the intensity of several primary emotions. |  |
| Lakhani et al. (2020) | Australia | Group 1: 10  Group 2: 14 Total: 24 | SCI-patients randomly assigned to one of two groups:  Group 1:  10 men (100%) 0 women (0%) Mean age: 56.20±20.74.  Group 2: 6 men (42.9%) 8 women (57.1%) Mean age: 48.00±16.21. | *Group 1:*  ASIA A: 7 (70%) ASIA B: 1 (10%) ASIA C: 2 (20%)  C1-C3: 2 (20%) C4-C5: 3 (30%) C6-C8: 2 (20%) T1-T6: 2 (20%) T12-L1: 1 (10%)  Traumatic: 9 (90%) Non-traumatic: 1 (10%)  Time since injury (d): 135.20±63.64  *Group 2:*  ASIA A: 3 (21.4%) ASIA C: 7 (50%) ASIA D: 4 (28.6%)  C4-C5: 3 (21.4%) C6-C8: 3 (21.4%) T1-T6: 1 (7.1%) T7-T11: 2 (14.3%) T12-L1: 5 (35.7%)  Traumatic: 8 (57.1%) Non-traumatic: 6 (42.9%)  Time since injury (d): 127.21±79.51 | Clinical sample (inpatients). Intervention performed in-hospital | Pilot crossover randomized controlled trial | Oculus Go VR headset | Patient Health Questionnaire-8 (PHQ-8)  Participants completed 3 feeling intensity scales prior to and following each VR session. Feeling intensity scales were adapted from the Depression Intensity Scale Circles. |  |
| Nunnerley et al. (2017) | New Zealand | Total: 12 (SCI: 5) | 4 men (80%) 1 woman (20%) Mean age: 46.2  Age range: 32-52 | Mean years living with SCI: 22  Range of years living with SCI: 10-30  No further information on injury characteristics | Community sample. Intervention performed in-center | Feasibility study | Oculus Rift headset and a Dynamic Control wheelchair joystick | Patient-reported outcomes |  |
| Pais-Vieira et al. (2022) | Portugal | 1 | 52-year-old man | SCI-patient with a 5-year history of back pain following surgery. ASIA complete T4 SCI stabilized.  32 years post-injury | Community sample. Intervention performed in-center | Single-case experimental design. | HTC VIVE Pro Eye | Patient-reported outcomes  The participant was asked about potential side effects due to  interaction with the VR set up. |  |
| Pais-Vieira et al. (2024) | Portugal | 1 | 52-year-old man | AIS A complete lesion at the T4 level 32 years post-injury | Community sample. Intervention performed in-center | Single-subject, longitudinal case study. | HCT VIVE Pro Eye | Embodiment Questionnaire, the Faces Pain Scale, the Verbal Pain Intensity Scale,  the Visual  Analogue Scale (VAS), and the Simulator  Sickness Questionnaire  Patient-reported outcomes. |  |
| Riva et al. (2000) | Italy | 1 | 26-year-old man | Complete paraplegia  4 years post-injury | Community sample Intervention performed in-center | Single subject case report and feasibility trial. | Thunder 400/C VR system | Patient-reported outcomes. |  |
| Tamplin et al. (2020) | Australia | Phase One: 6  Phase Two: 6  Total: 12 | Phase One: 6 men (100%) Mean age: 49.33. Age range: 26-70.  Phase Two: 5 men (83.33%) 1 woman (16.67%) Mean age: 47.5. Age range: 26-68.  Total: 11 men (91.7%) 1 woman (8.3%)  Mean age: 48.4. Age range: 26-70. | Phase One: C7A, C5A, T10A, T1A, C5B (n=2)  4 cervical 2 thoracic  Phase Two: T2A, T6A, C4D, T4A, T3C, C2D 2 cervical 4 thoracic  Total:  6 cervical 6 thoracic | Inpatients recruited from the Victorian Spinal Cord Service. Intervention performed in-facility | Non-randomized feasibility design with a two-phase iterative approach | Oculus Rift  HTC VIVE,  Samsung Gear VR | Patient-reported outcomes.  Psychosocial Impact of Assistive devices Scale (PIADS)– self-esteem domain. |  |
| Trost et al. (2022) | International (USA and Australia) | Interactive condition: 17  Passive condition: 10  Total: 27 | Interactive condition: 16 men (94.1%) 1 woman (5.9%) Mean age: 45.8  779  Passive condition: 6 men (60%) 4 women (40%) Mean age: 36.8  Total: 22 men (81.48%) 5 women (18.52%) Mean age: 42.5 Age range: 42.5 | Interactive condition:  T6, T9, T7-12, T7(x5), T4, T12(x3), C7-T1, T10-12, T1, T10, T11-12 16 thoracic 1 thoracic-cervical Time since injury: 2-39 years (mean: 13.7)  Passive condition:  T5, T4, T7-9, T3, T11(x2), T6, T12, T8-9, T10 10 thoracic  Time since injury: 1-15 (mean: 7.0)  Total: 26 thoracic 1 thoracic-cervical 0 cervical  Time since injury: 1-39 years (mean: 11.22) | Community sample. Conducted at home | Non-randomized, controlled, single-blind pilot study design. | HTC VIVE | Positive and Negative Affect Schedule (PANAS) – presession to postsession  Patient  Health Questionnaire-9 (PHQ-9) depression item – preintervention to postintervention |  |
