# Supplementary material for: Psychological outcomes of extended reality interventions in spinal cord injury rehabilitation: a systematic scoping review
Source: Spinal Cord. 2025 Jan 9;63(2):58–65. doi: 10.1038/s41393-024-01057-7 (PMC11810788; doi:10.1038/s41393-024-01057-7)
Supplement: Supplementary file 3 — Supplement 3. Extraction of intervention and relevant main results [file 41393_2024_1057_MOESM3_ESM.docx]

| Author (year) | Intervention | Relevant Main Results |
| --- | --- | --- |
| Austin et al. (2021) | All participants attended a single session scheduled at the same time of day to control for circadian effects on wakefulness in individuals with SCI. Baseline neuropathic pain (NP) levels were assessed using an 11-point numerical rating scale, measuring average, worst, least, and current NP intensity over the preceding week. The study examined the effects of two VR devices—3D head-mounted display (HMD; Oculus Rift) and 2D screen (laptop)—using identical software (‘Nature Trek’) for 15 minutes per intervention. Post-intervention NP scores were analyzed immediately following each session. A 60-minute washout period between interventions was implemented to prevent carryover effects. All assessments and VR sessions were conducted in a temperature-controlled environment. Participants also reported any discomfort with the headset or symptoms of cybersickness before, during, or after using the 3D HMD device. | Baseline mean scores for the DASS-21 subscales were within the normal clinical range (stress: mean 5.56 ± 2.92 SD; anxiety: mean 3.19 ± 2.79 SD; depressive mood: mean 2.69 ± 2.93 SD). Post-intervention, no significant differences were observed between the 3D HMD and 2D screen conditions in changes to the DASS-21 subscale scores. Moreover, linear mixed-model analysis indicated that neither the sense of presence nor the type of VR device significantly influenced mood levels. |
| Azurdia et al. (2022) | Participants completed three sessions, each separated by a minimum of one week, with the order of treatments randomized. During the initial session, participants were introduced to the HTC Vive head-mounted display (HMD). In the second session, they were randomly assigned to either a VR or non-VR condition, with the remaining condition completed during the third session. Each session included a 6-minute sub-maximal exercise trial on a standard arm cycle ergometer under the assigned condition. In the VR condition, participants navigated a self-selected virtual environment while pedaling an arm ergometer simulating forward motion at a cadence of 50 (±5) RPMs. Exercise intensity was adjusted to achieve a heart rate between 60% and 70% of the age-predicted maximum for those with low-level paraplegia or a Borg Rating of Perceived Exertion (RPE) score of 11–15 for those with tetraplegia or high-level paraplegia. Following the VR condition, participants completed questionnaires and a 10–15-minute semi-structured interview to reflect on their experience and the perceived impact of VR on their exercise session. | PSEQ:   \| **Group** \| **Value** \| **95% CI** \| **P** \| **Cohen’s d** \| **SE** \| \| --- \| --- \| --- \| --- \| --- \| --- \| \| VR \| 1.82±1.60 \| 0.74-2.9 \| 0.009 \| 1.59 \| 0.48 \| \| Non-VR \| 4.10±1.22 \| 3.3-4.9 \|  \|  \| 0.36 \|   FSS:   \| **Group** \| **Value** \| **95% CI** \| **P** \| **Cohen’s d** \| **SE** \| \| --- \| --- \| --- \| --- \| --- \| --- \| \| VR \| 2.36±2.25 \| 0.85-3.8 \| 0.012 \| 1.19 \| 0.68 \| \| Non-VR \| 5.10±2.30 \| 3.5-6.6 \|  \|  \| 0.69 \|   Participants shared that they were able to divert their attention from pain during exercise, as reflected in statements such as: “I wasn’t focusing on my body. Just seeing where I was and enjoying the trip, it just was not so hard; I wasn’t thinking of the pain. The pain wasn’t even on my mind.”  They also noted enhancements in their mental state, describing the experience as enjoyable and time-passing unexpectedly quickly. For example: “I was surprised at how fast it went by; I still thought I had way more time than I normally would have,” and “Using virtual reality really reduced the pain and makes you think… I can do this again.”  Additionally, participants reported feeling more motivated and determined to engage in exercise, attributing this to VR making the activity more enjoyable and manageable. When asked about VR's impact on exercise adherence, one participant remarked: “Yes, because exercise is boring, and I feel like I could definitely get into it. It’s still all new to me, I’m still wowing about the experience, but I think it would definitely encourage me; it would be way more exciting and interesting.” |
| Chu et al. | Virtual reality-based arm and leg cycling sessions began on the same day as the completion of routine rehabilitation training. The arm and leg cycling device (DN-813) was connected to VR glasses (Lei Niao Air Plus) via a sensor interface. The patient actively controlled the device to coordinate upper and lower limb movements, while a virtual character mirrored their cycling activity on a simulated road. Notably, the synchronization between the sensor and the character’s pedaling actions, lap counts, and frequency displayed in the VR glasses was seamless.  The therapist had the capability to independently adjust elements of the virtual environment, such as the time of day (e.g., sunrise, noon, sunset) and weather conditions (e.g., sunny, cloudy, snowy), providing a dynamic and engaging simulation. The addition of virtual participants created a competitive atmosphere, encouraging the patient to increase their physical activity. Real-time feedback on exercise metrics—such as distance covered, duration, calories burned, and laps completed—was displayed in the upper section of the virtual interface. This feedback allowed patients to set personal daily exercise targets, fostering motivation and adherence to their exercise routines.  For simultaneous treatment, transcutaneous spinal cord stimulation (tSCS) was applied using biphasic rectangular pulses of 0.2 ms at a frequency of 30 Hz for 30 minutes. Virtual reality-based arm and leg cycling sessions, combined with tSCS, were conducted for 30 minutes per session, once a day, five days a week, over a six-week period. | As somatic functioning improved, there was a significant reduction in anxiety and depression levels. |
| Donati et al. (2016) | The protocol consisted of six components:   1. An immersive VR environment where seated patients used their brain activity, recorded via a 16-channel EEG, to control a human body avatar, with visuo-tactile feedback provided. 2. The same virtual environment and brain-machine interface (BMI) protocol, but with patients positioned upright using a stand-in-table device. 3. Training on a robotic body weight support (BWS) gait system on a treadmill. 4. Training on an overground track using a fixed BWS gait system. 5. Training on a treadmill with a brain-controlled robotic BWS gait system. 6. Gait training using a brain-controlled robotic exoskeleton equipped with 12 degrees of freedom and sensorized for enhanced functionality.   In all components except 3 and 4, patients received continuous tactile feedback from virtual or robotic devices through a haptic display applied to the skin on their forearms. Activity complexity was progressively increased to promote cardiovascular stability and improved postural control. In addition to routine clinical evaluations conducted before and after each activity, comprehensive clinical assessments were periodically performed to monitor changes in neurological status, as well as psychological and physical conditions, in patients with SCI. | All patients demonstrated high levels of emotional stability and achieved favorable scores in assessments of quality of life, depression, and self-esteem, with minimal variation observed over the course of the study. Psychological support was provided as needed based on individual requirements; however, no psychiatric medication was necessary. |
| Ferrero et al. (2023) | After the experimental setup was validated with able-bodied participants, it was tested with individuals with SCI. These participants attended five sessions on separate days, each consisting of three phases: VR, calibration, and closed-loop control of an exoskeleton.  Each session began with a brief period where participants were not required to perform any mental tasks. An acoustic cue then signaled the start of the first mental task, during which participants were instructed to relax in an idle state. Another cue indicated the transition to the motor imagery (MI) phase, where participants imagined leg movements as if walking. Finally, a third cue signaled the regressive count task, where participants performed mental math subtractions. Only two data classes were analyzed: "Idle state" and "MI of gait."  For participants with SCI, VR sessions were shorter compared to able-bodied participants. They completed six VR trials: three where the avatar remained static and three where the avatar walked through a corridor. Calibration involved 12 trials, and closed-loop control of the exoskeleton consisted of seven trials per session. During these sessions, researchers monitored participants’ perceived exertion levels.  At the conclusion of the final session, participants completed two questionnaires: the NASA Task Load Index, which assessed perceived mental workload, and the Quebec User Evaluation of Satisfaction with Assistive Technology, which evaluated their satisfaction with the exoskeleton. | Patient P1 reported higher overall satisfaction with the exoskeleton compared to Patient P2. Both patients agreed that the device was not particularly comfortable, and P2 noted that adjustments to the exoskeleton were challenging. Despite finding the process physically and mentally demanding, both patients expressed satisfaction with their performance outcomes.  NASA-TLX:  P1 Mental demand: 85/100  P2 Mental demand: 80/100  P1 Frustration level: 90/100  P2 Frustration level: 0/100 P1: Temporal demand: 70/100 P2: Temporal demand: 10/100  QUEST: P1: How comfortable the device is? 3/5 P2: How comfortable the device is? 3/5  P1: How easy is it to use the device? 4/5 P2: How easy is it to use the device? NA |
| Flores et al. (2018) | Participants completed pre-treatment psychological assessments before engaging in VR-based DBT skills training. Using Oculus Rift DK2 goggles, they experienced a 3D virtual environment simulating a serene river surrounded by trees, mountains, and ambient nature sounds.  Patient 1, unable to move his head due to a breathing tube, had the goggles held near his face by a therapist, while Patient 2 wore the goggles and could explore the VR world visually. During the sessions, participants listened to one of three mindfulness audio tracks synchronized with the VR environment:   1. **Observing Sound (8.5 mins):** Guided participants to focus on and observe sounds, such as a bell's tones, while maintaining awareness of their breath. 2. **Observing Visuals (10 mins):** Encouraged observing the visual details of the VR river environment and gently refocusing if the mind wandered. 3. **Wise Mind (8 mins):** Invited participants to imagine themselves as part of the river’s flow, fostering self-awareness and calmness.   Patient 1 completed four sessions, cycling through all three tracks, with "observing visuals" repeated. Patient 2 attended two sessions—"observing visuals" and "observing sound"—before being discharged due to health improvements. | Patient 1 reported reductions in feelings of depression, anxiety, and emotional upset following VR DBT sessions. On Study Day 1, their pre-session depression rating on a 0-10 GRS scale dropped from 8 ("pretty depressed") to 6 ("moderately depressed"), anxiety decreased from 8 to 6, and emotional upset reduced from 6 to 5. These outcomes aligned with predicted decreases in negative primary emotions after the intervention.  Patient 2 also experienced improvements on Study Day 1, with depression ratings dropping from 6 to 5, anxiety from 7 to 6, and emotional upset from 9 ("severe") to 6 ("moderate"). On their second session, depression decreased from 3 to 2, anxiety from 4 to 3, but emotional upset remained unchanged at 3. Patient 2 showed significant reductions in ASD/PTSD symptoms and lower fear and shame ratings after the first session. However, sadness, anger, and guilt ratings were unexpectedly higher immediately post-session. On Day 2, their ratings of negative emotions were minimal (zero or near zero) both before and after treatment. Due to medical improvements, Patient 2 was discharged after Day 2.  Both patients found the intervention engaging and beneficial. They noted that VR helped them focus, practice mindfulness, and saw potential for VR-based DBT skills training to benefit future spinal injury patients. |
| Lakhani et al. (2020) | Participants were divided into two groups: Group 1 underwent the treatment condition in week 1 and the control condition in week 2, while Group 2 followed the reverse order.   - **Control condition:** Participants engaged in standard rehabilitation activities tailored to their individual goals, including daily occupational and physiotherapy sessions, with support from social work, psychology, leisure therapy, and nursing professionals. - **Treatment condition:** Alongside their regular rehabilitation, participants attended three additional 20-minute VR sessions using the Oculus GO headset. Each session featured diverse virtual natural environments:   - **Session 1:** A dive in the Great Barrier Reef.   - **Session 2:** Exploration of an oil rig ecosystem, observing veterinarians in Borneo, and visiting Bryce Canyon and Joshua Tree National Park.   - **Session 3:** Virtual trips to Victoria Falls, an underwater national park, and an Antarctic dive to view leopard seals. | After each VR session, participants reported significantly improved happiness, mood, and calmness. Both groups showed lower PHQ-8 scores after the treatment condition, indicating improved psycho-emotional health.  A t-test revealed a significant difference in Td1 PHQ-8 scores between groups, with Group 1 (intervention condition) showing greater improvement compared to Group 2 (control condition). However, a second t-test found no significant difference in Td2 PHQ-8 scores between groups. While Group 2 (intervention condition) exhibited improved psycho-emotional health compared to Group 1 (control condition) during Td2, the change was not statistically significant. |
| Nunnerley et al. (2017) | A Wheelchair Training System was developed using an Oculus Rift headset and a Dynamic Control wheelchair joystick. The 3D simulation replicated the TransitioNZ unit at the Burwood Spinal Unit, a transitional rehabilitation space for patients with SCI during their final four weeks before discharge. This semi-independent environment includes private ensuited bedrooms, a shared living area with a lounge, kitchen, and outdoor space.  Participants trialed the system in small groups of two to three people while remaining in their own wheelchairs. Each session lasted 20–30 minutes. Participant feedback was captured through digital recordings and field notes during the trials.  Follow-up focus groups and individual interviews were conducted within one to two weeks of the VR trials at Burwood Hospital. Sessions, lasting 40–50 minutes, included both experienced wheelchair users and clinicians. Semi-structured questions explored participants’ experiences with the equipment, as well as its design, practicality, and potential applications. The same questions were used for both focus groups and individual interviews. | Experienced wheelchair users reflected on the anxiety they felt about an uncertain future following their SCI and highlighted how the VR system could help: *“People can be concerned about the unknown and there’s lots of unknowns when you come into a spinal unit and there’s lots of life changes, so by having to experience something before you actually physically do it, in that regard, it would probably be really helpful.”*  Both groups agreed that the VR system could help reduce fears about navigating community environments and boost confidence in managing obstacles with a wheelchair. One participant noted: *“Those very first one or two times it just, you know, gives you the confidence, perhaps, instead of taking a week to getting used to yourself being on your own in a wheelchair or being able to go around, you know, the spinal unit, in your wheelchair, it would give you the confidence, that might shorten to one or two days. I think it would just build your confidence earlier. The more you can build someone’s confidence earlier on in the piece, the better it’s gonna be.”*  Another participant added: *“I think it would just particularly help in those early days when perhaps you can’t go to the gym or you can’t do too much but you can sit on the simulator for half an hour and whizz around and you know, that would a) build your confidence but b) you know, you’d start to think of questions and you know, little things too.”*  One individual highlighted the system’s engaging nature: *“Without trivialising it ... it’s kind of fun, which is kind of a nice aspect when you’re in rehab and you’ve been through a really full-on traumatic injury and it’s quite a heavy thing, ... it’s a novel approach, can make things more interesting.”* |
| Pais-Vieira et al. (2022) | Ten sessions were conducted twice weekly, each lasting 70–90 minutes. Sessions included:   1. **Initial evaluation (10 minutes):** Assessing equipment comfort and gathering feedback on the previous session, pain, and stress levels. 2. **VR interaction (20–25 minutes):** Divided into habituation, EEG baseline, and real-time neural data acquisition. 3. **Questionnaires:** Followed by setup and debriefing.   Participants used a VR headset with embedded headphones and hand controllers. During habituation, they chose one of 16 scenarios featuring diverse landscapes (grass, sand, stone, water) and interacted by triggering avatar steps. The avatar was viewed in first-person, showing arms, torso, legs, and feet.  In the baseline phase, 20 seconds of neural activity were recorded with eyes open in the chosen scenario. During neural data acquisition, visual cues—gray ("Trial starting"), green ("Walk"), and red ("Stop")—guided participants through 40 trials per session (20 "Walk" and 20 "Stop"). Upon green cues, participants imagined lifting and stepping with alternating legs; for red cues, they imagined standing still and enjoying the scenario. | The participant reported only mild general discomfort in 2 out of 10 sessions (sessions 1 and 5), with no discomfort in the remaining sessions.  Overall, the participant described the experience positively, sharing statements such as, *“there are not enough stars in the universe to rate how much I am enjoying this experience,”* and, *“this is the first time that I feel like I am standing up and walking in 30 years.”*  In three sessions involving water scenarios, the participant noted feeling a cold sensation in his legs upon entering the VR environment, including sessions without the thermal-tactile sleeve. He described the sensation as pleasant and surprising, explaining that he had not felt cold in his legs or feet for 30 years but did not find it uncomfortable. |
| Pais-Vieira et al. (2024) | The intervention consisted of two phases over 14 months:   1. **Phase 1:** Ten biweekly sessions (S1–S10). 2. **Phase 2:** Twenty-four weekly sessions (S11–S34), followed by clinical assessment.   Each session, lasting 70–90 minutes, was conducted by a team of 1–4 researchers and included:   - **10 minutes:** Comfort evaluation, feedback on the previous session, and discussion of pain/stress levels. - **20–25 minutes:** VR interaction, divided into habituation, EEG baseline (30 seconds of neural activity recording with eyes open), and neural data acquisition with real-time decoding. - **Remaining time:** Questionnaire completion, setup, and debriefing.   Participants used a VR headset with headphones and hand controllers to interact with 16 scenarios featuring various landscapes (e.g., grass, sand, stone, water). Scenarios and avatar appearances were chosen by participants, who viewed their avatar from a first-person perspective.  During neural data acquisition, colored cues guided actions: gray (trial start), green ("Walk"), and red ("Stay still"). In 40 trials per session (20 "Walk" and 20 "Stay still"), participants imagined stepping or remaining still based on the cue. In S1–S10, no performance feedback was given; the avatar moved automatically with a slight delay, independent of neural activity, to avoid negative reinforcement. Feedback was introduced in S11–S34, using high-pitch sounds for correct neural decoding and low-pitch sounds for incorrect decoding. | When asked about the intervention’s impact on his well-being and quality of life, during both session interviews and pre/post-intervention assessments, the patient consistently expressed enthusiasm for continuing the sessions beyond the study. He highlighted that *“the sensation of standing and walking through the scenarios”* offered relaxation and well-being, contributing to an improved quality of life.  S1-S10. Stress (no), engagement (yes)  S11. Stress (yes), engagement (yes)  S12. Stress (no), engagement (yes)  S13. Stress (no/yes), engagement (yes)  S14. Stress (no), engagement (yes)  S15. Stress (yes), engagement (yes)  S16. Stress (yes), engagement (yes)  S17. Stress (yes), engagement (no)  S18. Stress (no/yes), engagement (yes)  S19. Stress (yes), engagement (yes)  S20. Stress (no), engagement (yes)  S21. Stress (no), engagement (yes)  S22. Stress (no) engagement (yes)  S23. Stress (no/yes?), engagement (yes)  S24. Stress (no), engagement (yes)  S25. Stress (no), engagement (yes)  S26. Stress (yes), engagement (yes)  S27. Stress (yes), engagement (yes)  S28. Stress (no), engagement (yes)  S29. Stress (yes), engagement (no/yes)  S30. Stress (no), engagement (yes)  S31. Stress (no), engagement (yes)  S32. Stress (no), engagement (yes)  S33. Stress (yes?), engagement (no?)  S34. Stress (no/yes?), engagement (no?) |
| Riva et al. (2000) | Researchers developed a prototype orthopedic appliance combining a semi-rigid, gait-inducing exoskeleton for upper body and lower limb support with a VR system. Designed for customizable rehabilitation, the device simulates the experience of walking along a mountain path.  The exoskeleton features a compressed-air-operated sling with microcylinders that replicate human gait, controlled by a two-button interface on the framework grips. Pressing the left button moves the left leg forward, while the right button moves the right leg. The framework includes wheels for mobility and houses the VR system and compressed-air mechanism. The VR environment uses real Alpine imagery and natural sounds to enhance realism, synchronized with the patient’s steps.  Two experimental sessions were conducted, each including two 15-minute trials separated by a 10-minute break. In each trial, the patient walked a virtual mountain path. During the second trial, a virtual runner was introduced to create a competitive element. Before and after each session, the patient rated his emotional and physical state using a questionnaire with 20 bipolar adjective pairs across six intensity levels. | Adjective ratings indicated slight improvements in self-confidence, willpower, relaxation, and activity levels. Nicola also reported subjective enhancements in well-being, mood, and sleep quality. These improvements were reflected in higher scores for mood-related adjectives (e.g., *likeable-unlikeable*, *happy-unhappy*) and state-related adjectives (e.g., *fast-slow*, *dynamic-static*). |
| Tamplin et al. (2020) | **Phase 1:** Participants trialed various VR headsets using *vTime*, a social VR app allowing up to four users (split into two groups of three participants plus a therapist) to meet in virtual spaces such as a campfire or tropical island. Although in separate rooms within the same building, the setup simulated a home-like low-latency broadband connection. Participants interacted as avatars, hearing each other via headphones and singing familiar choruses from memory. Researchers evaluated the experience using questionnaires and semi-structured interviews, analyzing responses through thematic analysis guided by the question: *“How do people with a spinal cord injury experience virtual reality and online singing groups?”*  **Phase 2:** Based on Phase 1 feedback, researchers developed a custom VR application featuring on-screen song lyrics and gaze-based controls for accessibility. Participants compared VR music therapy to face-to-face and teleconferenced group singing.   - **Face-to-face:** Participants sang together with a music therapist, selecting songs from a six-song list. - **Teleconference:** Participants sang via Zoom while in separate rooms, guided by the music therapist. - **VR:** Using the same song list and avatars, participants sang together in a virtual space with the therapist.   As in Phase 1, questionnaires and interviews were used to assess the user experience across all modalities. | **Phase 1:** The perceived psychosocial impact of VR devices was rated as “somewhat positive,” with a median PIADS self-esteem subscale score of 0.56 (0.13–2.25).  **Phase 2:** The psychosocial impact of the VR headset and platform also indicated a “somewhat positive” effect, with a higher median PIADS self-esteem subscale score of 2.06 (0.13–2.75).  **Thematic Analysis Themes:**  a) **Positive Experience:** Participants described VR as “interesting,” “fun,” “cool,” and “different.”  b) **Immersive and Transportative:** VR provided a temporary escape from reality and a distraction from injury or pain.   - *“It just distracted me, I guess, from my injury and current situation, momentarily”* (Pt 4). - *“It was a release from reality... it took you into a world of fantasy”* (Pt 6).   c) **Reduced Singing Inhibitions:** VR created a sense of anonymity, helping participants feel less self-conscious about singing.   - *“You’ve sort of got that invisible mask where you can make an idiot out of yourself”* (Pt 3).   d) **Reduced Social Cues:** Some participants felt VR lacked human interaction cues, impacting timing and connection.   - *“One of the difficulties... is that it’s so divorced of any kind of human cues that you get”* (Pt 1).   e) **Comfort and Accessibility:** The VR equipment was reported as comfortable, accessible, and easy to use. |
| Trost et al. (2022) | Participants completed baseline measures of neuropathic and non-neuropathic pain intensity, pain quality and interference, and depressive symptoms (NRS, NPS, and PHQ-9). The 10-day intervention was conducted at participants’ homes, with a research assistant present.  **Interactive Condition:** Participants used the HTC Vive for the VRWalk intervention, with wireless controllers tracking arm movements and a head-mounted display (HMD) providing a first-person view of their avatar. The avatar was customized to match participants’ characteristics, and gameplay emphasized exploration in open virtual worlds. Participants collected virtual coins during gameplay to earn up to $75 as motivation. Progress was saved across sessions for continuity.  **Passive Condition:** Participants also selected customized avatars but had no control over the virtual environment. Instead, they observed pre-recorded first-person virtual walking. To maintain consistency, participants could also earn up to $75 for session completion.  **Intervention Details:** Participants completed 20 sessions over 10 days, with two 30-minute sessions daily, spaced at least four hours apart. VR immersion was limited to 5 minutes per session, with breaks allowed. Pain and affect measures (NRS and PANAS) were recorded before and after each session. Follow-up measures, including NRS, NPS, PHQ-9, PGIC, TEI, and embodiment-specific questions, were collected 7 days after the intervention, with a second NRS follow-up for average neuropathic pain intensity conducted 2 weeks later. | A significant **Time × Condition interaction** was found for changes in positive affect from pre- to post-session, *F*(1,25) = 9.54, *P* < 0.01. Follow-up analyses showed a significant increase in positive affect for the interactive condition, *F*(1,16) = 48.63, *P* < 0.001, and a marginal increase for the passive condition, *F*(1,9) = 4.23, *P* = 0.07. Negative affect significantly decreased from pre- to post-gaming, *F*(1,25) = 4.52, *P* < 0.05.  No significant main effects were observed for session timing (early vs. late) on pain, *F*(1,25) = 1.47, *P* = 0.24, positive affect, *F*(1,25) = 2.79, *P* = 0.11, or negative affect, *F*(1,25) = 1.65, *P* = 0.21. Additionally, no significant interactions between session timing and condition were found for positive affect, *F*(1,25) = 0.45, *P* = 0.51, or negative affect, *F*(1,25) = 0.70, *P* = 0.41.  Depressive symptoms significantly declined across the study, regardless of condition, *F*(1,25) = 7.44, *P* < 0.05.  Positive affect (PANAS) INT: pre= 24.35(7.40), post= 26.61 (6.45) [increase] Positive affect (PANAS) PAS: pre=35.00(6.05), post=35.96 (6.32) [increase] Negative affect (PANAS) INT: pre= 10.95 (1.28), post= 10.72 (1.07) [decrease] Negative affect (PANAS) PAS: pre= 10.78 (1.97), post= 10.41 (1.00) [decrease]  Depression (PHQ-9) INT: pre= 6.50(5.38), post=5.19 (4.40) [decrease] Depression (PHQ-9) PAS: pre= 10.20 (6.29), post= 8.90 (6.35) [decrease] |
