# Supplementary material for: Psychological outcomes of extended reality interventions in spinal cord injury rehabilitation: a systematic scoping review
Source: Spinal Cord. 2025 Jan 9;63(2):58–65. doi: 10.1038/s41393-024-01057-7 (PMC11810788; doi:10.1038/s41393-024-01057-7)
Supplement: Supplementary file 4 — Supplement 4. Risk of bias assessment, including the foremost quality concerns, of the 13 included studies [file 41393_2024_1057_MOESM4_ESM.docx]

| Author(s) | Risk of bias assessment | Foremost quality concerns |
| --- | --- | --- |
| Austin PD, Craig A, Middleton JW, Tran Y, Costa DSJ, Wrigley PJ, et al. | Fair | Participants and researchers delivering the interventions were aware of the assigned intervention during each period of the trial; Assessment of the outcome could have been influenced by knowledge of the received intervention. |
| Azurdia D, Acuña SA, Narasaki-Jara M, Furtado Jr O, & Jung T. | Poor | Participants and researchers delivering the interventions were aware of the assigned intervention during each period of the trial; Assessment of the outcome could have been influence by knowledge of the received intervention; No information about whether the data that produced the result was analysed in accordance with a pre-specified analysis plan that was finalized before unblinded outcome data were available for analysis; The numerical result being assessed is likely to have been selected, on the basis of the results, from multiple eligible outcome measurements (i.e., scales) within the outcome domain; No information provided to assess risk of bias arising from period and carryover effects. |
| Chu X, Liu, S, Zhao X, Liu T, Xing Z, Li Q, et al. | Good | Uncertain as to whether the patient’s history was clearly described and presented as a timeline; No adverse events (harms) or unanticipated events were identified and described. |
| Donati ARC, Shokur S, Morya E, Campos DSF, Moioli RC, Gitti CM, et al. | Fair | Not reported whether all eligible participants that met the prespecified entry criteria were enrolled; Study size not sufficiently large enough to provide confidence in the findings; Not reported as to whether those assessing the outcomes were blinded to the participant’s intervention; No statistical methods examining changes in outcome measures from before to after the intervention for our objects of interest specifically; No statistical tests that provided *p* values for the pre-to-post changes for our objects of interest specifically. |
| Ferrero L, Quiles V, Ortiz M, Iáñez E, Gil-Agudo Á, & Azorín JM | Poor | Eligibility criteria/selection for the study population was not prespecified and clearly described; Unable to determine whether participants in the study were representative of those who would be eligible for the intervention in the general or clinical population of interest; Not reported as to whether all eligible participants that met the prespecified criteria were enrolled; Those assessing the outcomes were not blinded to the participants’ intervention; No statistical methods examining changes in outcome measures from before to after the intervention for our objects of interest specifically; No statistical tests that provided *p* values for the pre-to-post changes for our objects of interest specifically; Outcome measures of interest were not taken multiple times before the intervention and multiple times after the intervention (i.e., no interrupted time-series design). |
| Flores A, Linehan MM, Todd SR, & Hoffman HG | Fair | Not reported whether the cases were consecutive; Cannot determine if the cases are comparable; Inadequate length of follow-up; Cannot determine whether the statistical methods are well-described. |
| Lakhani A, Martin K, Gray L, Mallison J, Grimbeek P, Hollins I, et al. | Fair | There exists potential that baseline differences between intervention groups at the start of the first period suggested a problem with the randomization process; The number of participants allocated to each of the two sequences was considered probably not equal, or nearly equal; No further information available to assess risk of bias concerning period and carryover effects; Participants were aware of their assigned intervention during each period of the trial, as too were those delivering the intervention; Data for a relevant outcome were possibly not available for all, or nearly all, participants randomized. There is probably insufficient evidence to prove that the result was not biased by missing outcome data, and it’s possible that missingness in the outcome depended on its true value; Outcome assessors were aware of the intervention received by study participants; Assessment of the outcome could potentially have been influenced by knowledge of the intervention received; It is potentially likely that assessment of the outcome was influenced by knowledge of the intervention received; There is no information reported to assess whether risk of bias is present in the selection of the reported result (e.g., whether the numerical result being assessed was likely to have been selected on the basis of the results from multiple eligible outcome measurements within the outcome domain, or multiple eligible analyses of the data) |
| Nunnerley J, Gupta S, Snell D, & King M | Good | Unable to tell whether the relationship between the researcher and the participants has been adequately considered. |
| Pais-Vieira C, Gaspar P, Matos D, Alves LP, da Cruz BM, Azevedo MJ, et al. | Good | The patient’s history was not clearly described and presented as a timeline; Lack of information about the participant pre-admission; No information about family and psychosocial history. |
| Pais-Vieira C, Figueiredo JG, Perrotta A, Matos D, Aguiar M, Ramos J, et al. | Good | The patient’s history was not clearly described and presented as a timeline; No information about family and psychosocial history. |
| Riva, G | Fair | The patient’s history was not clearly described and presented as a timeline; Diagnostic tests and assessment methods were not clearly described. |
| Tamplin J, Loveridge B, Clarke K, Li Y, & Berlowitz DJ. | Fair | No multiple measurements of the outcome pre- and post- the intervention; Unclear as to whether participants included in any comparisons were receiving similar treatment/care, other than the exposure or intervention of interest. |
| Trost Z, Anam M, Seward J, Shum C, Rumble D, Sturgeon J, et al. | Fair | No multiple measurements of the outcome pre- and post- the intervention for assessment of depression; Unclear as to whether participants included in any comparisons were receiving similar treatment/care, other than the exposure or intervention of interest. |
